# New Caries Diagnostic Tools in Intraoral Scanners: A Comparative In Vitro Study to Established Methods in Permanent and Primary Teeth

**DOI:** 10.3390/s22062156

**Published:** 2022-03-10

**Authors:** Maximiliane Amelie Schlenz, Berenike Schupp, Alexander Schmidt, Bernd Wöstmann, Ingo Baresel, Norbert Krämer, Nelly Schulz-Weidner

**Affiliations:** 1 Dental Clinic—Department of Prosthodontics, Justus Liebig University, Schlangenzahl 14, 35392 Giessen, Germany; berenike.schupp@dentist.med.uni-giessen.de (B.S.); alexander.schmidt@dentist.med.uni-giessen.de (A.S.); bernd.woestmann@dentist.med.uni-giessen.de (B.W.); 2 Dental Practice Drs. Baresel, 90556 Cadolzburg, Germany; ingo.baresel@t-online.de; 3 Dental Clinic—Department of Pediatric Dentistry, Justus Liebig University, Schlangenzahl 14, 35392 Giessen, Germany; norbert.kraemer@dentist.med.uni-giessen.de (N.K.); nelly.schulz-weidner@dentist.med.uni-giessen.de (N.S.-W.)

**Keywords:** intraoral scanners, dental caries, diagnostic techniques, digital dentistry, pediatric dentistry, interdisciplinary study

## Abstract

The aim of this in vitro study was to systematically investigate new caries diagnostic tools, including three intraoral scanners, and compare them to established diagnostic methods. For a standardized analysis of occlusal and proximal caries lesions, human permanent and primary teeth (n = 64) were embedded in models and investigated in a phantom head using six different caries diagnostic methods: visual examination, bitewing radiography, Diagnocam (KaVo, Biberach, Germany), Trios 4 (3Shape, Copenhagen, Denmark), iTero Element 5D (Align Technology, San José, CA, USA), and Planmeca Emerald S (Planmeca, Helsinki, Finland). The diagnostic methods were investigated and compared to reference µ-CT for permanent and primary teeth separately. For occlusal caries diagnostics in permanent teeth, the best agreement to the reference (reliability) was obtained for Planmeca Emerald S (ĸ = 0.700), whereas in primary teeth, for visual examination (ĸ = 0.927), followed by Trios 4 (ĸ = 0.579). Regarding proximal caries diagnostics, bitewing radiography, as the gold standard, exhibited the highest agreement for permanent (ĸ = 0.643) and primary teeth (ĸ = 0.871). Concerning the analysis of the diagnostic quality (sensitivity and specificity) using receiver operating characteristic (ROC) curve analysis, comparable findings were obtained for area under curve (AUC) values as for reliability. No diagnostic method could be identified that is generally suitable for occlusal and proximal lesions in both dentitions. Overall, caries diagnostics with intraoral scanners seem to be interesting tools that should be further investigated in clinical studies.

## 1. Introduction

Today, intraoral scanners offer many additional applications beyond digital impression acquisition, including the determination of tooth color, treatment simulation, and the monitoring of tooth movement and wear [1,2,3,4]. Meanwhile, three commercially available intraoral scanners, including Trios 4 (3Shape, Copenhagen, Denmark), iTero Element 5D (Align Technology, San José, CA, USA), and Planmeca Emerald S (Planmeca, Helsinki, Finland), provide caries diagnostic tools for the detection of occlusal and/or proximal caries lesions integrated in their intraoral scanners [5]. Due to the fact that dental caries is still the most common non-communicable disease worldwide and remains a significant and costly public health issue [6,7], intraoral scanner-based diagnostics might help to detect and monitor enamel caries lesions at an early stage to enable minimally invasive treatment options [8,9,10]. Furthermore, caries experience and malocclusion are related [11]. Thus, the caries diagnostic tools involving intraoral scanners might also be useful for orthodontics, in particular in children from lower socio-economic backgrounds.

However, non-cavitated lesions are especially difficult to diagnose, and correct diagnostics are crucial for an optimized treatment decision [12]. False negative findings may result in caries lesions being detected too late, thus making minimally invasive treatment methods obsolete. In contrast, false positive results may cause unnecessary invasive treatments [13].

Several caries diagnostic methods are well described in the literature, but there is no universal diagnostic approach that is equally effective for occlusal and proximal caries lesions. While visual examination is highly recommended for the diagnosis of early occlusal caries lesions, proximal lesions cannot be analyzed due to the presence of adjacent teeth. Therefore, bitewing radiography is recommended as an adjunct to visual examination for the early detection and monitoring of proximal caries lesions over time, as well as advanced occlusal caries lesions in dentin [13,14,15].

Although bitewing radiography is described as the gold standard for proximal caries lesions, a clear disadvantage is the use of ionizing radiation, which limits the monitoring of caries lesions [16]. Thus, bitewing radiography is not suitable for pregnant women, and should be carefully used in children. Furthermore, bitewing radiography is not helpful for the detection of early occlusal caries lesions due to the superimposition of the enamel layer [17,18,19,20,21].

The Diagnocam (KaVo, Biberach, Germany) was developed to provide a radiation-free diagnostic method for caries diagnosis [22,23]. This method is used in particular for treating children and monitoring caries lesions at short intervals owing to the absence of ionizing radiation. Meanwhile, the Diagnocam has been established in several studies as an effective diagnostic tool for the detection of proximal caries lesions, whereas data for occlusal caries diagnostics are scarce. Additionally, it must be mentioned that Diagnocam is a separate appliance that cannot be integrated into other hardware or software solutions [23,24,25]. 

In addition to the Diagnocam, with its near-infrared transillumination technique, quantitative light-induced fluorescence (QLF), fiber-optic transillumination (FOTI), and electrical conductance (EC) have been described in the literature for the detection of early caries lesions [26]. However, these tools should be used to establish diagnostic methods for caries. A combination of different caries diagnostic methods is recommended as routine procedure before the invasive treatment of non-cavitated early caries lesions [13,21,26,27,28,29].

Considering these limitations of established caries diagnostic tools, the new intraoral scanner-based caries diagnostic tools might be a promising enhancement. Nevertheless, new diagnostic methods have to offer advantages without sacrificing diagnostic accuracy [30]. Therefore, systematic investigations are urgently needed to confirm their efficacy.

Although the potential benefit of intraoral scanners in this regard is mentioned in the literature [5], only three studies related to caries diagnostic methods have been published. Two studies investigated an experimental caries diagnostic tool integrated in the Trios 3 (3Shape), which is not commercially available, regarding occlusal caries diagnostics in vitro and in vivo [31,32]. The third clinical study compared the iTero Element 5D to established bitewing radiography showing a higher sensitivity for the detection of early proximal caries lesions with intraoral scanners [33].

To the best of our knowledge, no study has systematically examined the new caries diagnostic tools for all three commercially available intraoral scanners. Therefore, the aim of this in vitro study was to investigate the three new intraoral scanner-based caries diagnostic tools (Trios 4, iTero Element 5D, and Planmeca Emerald S) in comparison to the established methods (visual examination, bitewing radiography, and Diagnocam). It was hypothesized that there would be no difference between the caries diagnostic methods and the reference method µ-CT in terms of reliability (I), sensitivity and specificity (II), and logistic regression (III).

## 2. Materials and Methods

### 2.1. Study Design

In this comparative in vitro study, three new intraoral scanner-based (Trios 4, iTero Element 5D, and Planmeca Emerald S) and three established caries diagnostic methods (visual examination, bitewing radiography, and Diagnocam) were investigated. Because no data were available for the new intraoral scanner-based diagnostic methods, the investigation was designed as a pilot study in the framework of a “validation study” to prepare a clinical trial. Non-destructive micro-computed tomography (µ-CT, TomoScope XS-FOV, Werth Messtechnik, Giessen, Germany) was used as a reference method [34,35,36,37,38]. The evaluation of the occlusal and proximal caries diagnostics was carried out separately for permanent and primary teeth. 

### 2.2. Standardization, Calibration, and Blinding

To ensure comparable testing conditions and to obtain reproducible data according to predefined criteria, one dentist (B.S.) performed all caries diagnostic methods [39]. In advance of this study, experts in each field thoroughly trained the examiner. The training included a theoretical part in which the criteria for caries diagnosis were explained. A practical exercise was performed on a model of extracted permanent and primary teeth. For the three new intraoral scanner-based caries diagnostic methods, two experts for intraoral scanning (I.B. for iTero Element 5D, and M.A.S. for Trios 4 and Planmeca Emerald S) provided instructions. A specialist in pediatric dentistry (N.S.-W.), with 20 years of clinical experience, conducted the calibration on the three established caries diagnostic methods (visual examination, bitewing radiography, and Diagnocam). Thus, it was possible to determine intra-rater (consistent findings of each individual examiner) and inter-rater reliability (different examiners arrived at consistent diagnoses) [39]. The intensity of agreement between the examiner and instructor was, thus, nearly perfect, according to Landis and Koch [40] (Cohen kappa coefficient (κ) > 0.81). Because of the in vitro design of the study, it was viable that the examiner could pause for one week between each diagnostic method. All teeth were analyzed with the respective diagnostic method only. The pause should reduce the bias of recalling previous results. After two weeks of completing the six caries diagnostic examinations, reference data were generated by an independent institute (Werth Messtechnik) using micro-computed tomography (µ-CT). The µ-CT data were analyzed by a second examiner (M.A.S.) without any knowledge of the results of the diagnostic methods.

If provided by a manufacturer, intraoral scanners were calibrated according to the manufacturer’s instructions before scanning. A quadrant scan was performed following a standardized scan path, consisting of the occlusal surface, oral surfaces, and buccal surfaces [41].

### 2.3. Preparation of Test Models

To enable a standardized comparison between the different caries diagnostic methods, an experimental design along with a clinical close setup was conducted. Therefore, human teeth (n = 64) of the permanent (n = 51) and primary (n = 13) dentitions were collected and extracted for therapeutic reasons at the Department of Pediatric Dentistry, Giessen, Germany, between March and May 2020, with informed consent from the patients or legal guardians. The age of the patients ranged from 2 to 18 years. Only primary molars and permanent premolars and molars were included in this study, while incisors, canines, and crowned or filled teeth were excluded. The teeth revealed occlusal and/or proximal caries lesions, as well as apparent superficial sound surfaces. After extraction, the teeth were carefully cleaned and stored according to ISO/TS 11405 (first in 0.5% chloramine-T trihydrate solution (Lysoform, Berlin, Germany) for seven days, followed by distilled water for a maximum of six months with weekly water change) [42]. The study was approved by the local ethics committee of the Medical Faculty (Reg. No. 143/09).

**Figure 1:**
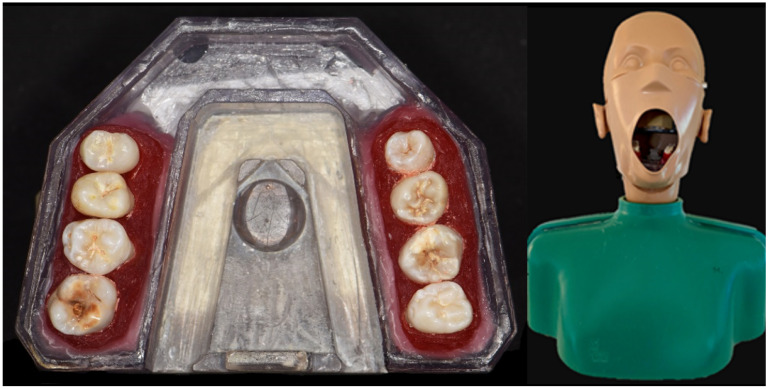
Example of test model (**left**) and test model mounted in a phantom head (**right**).

Eight teeth (four in each lateral tooth area) were embedded in pink-colored acrylic resin (Palavit G, Kulzer GmbH, Hanau, Germany) with proximal contact and mounted in a model base (A-REEM BGR1, Frasaco GmbH, Tettnang, Germany) made of low X-ray contrast plastic with recesses for placing digital X-ray sensors and a socket to screw the model into a phantom head (Figure 1).

### 2.4. Diagnostic Methods

All diagnostic methods were carried out with the respective model mounted on a phantom head, except for bitewing radiography. The ambient lighting of 500 lx was controlled using a luxmeter (LED Luxmeter HT 309, HT Instruments GmbH, Korschenbroich, Germany) [43,44].

**Figure 2:**
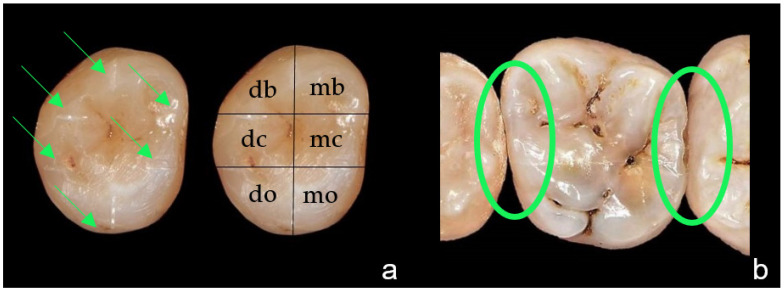
(**a**) Example of a tooth with prepared grooves (green arrows, left) and superimposed with a grid resulting in six areas: mesiobuccal (mb), distobuccal (db), mesiocentral (mc), distocentral (dc), mesio-oral (mo), disto-oral (do, right). (**b**) Example of a tooth with mesio-proximal and disto-proximal areas (green circles).

For standardized measurement, the occlusal surface of each tooth was divided into six areas: mesiobuccal, distobuccal, mesiocentral, distocentral, mesio-oral, and disto-oral [1]. Therefore, six grooves were prepared and served as reference points for the grid (Figure 2a) [45]. The proximal lesions were investigated in two areas: the mesio-proximal and disto-proximal surfaces (Figure 2b).

#### 2.4.1. Visual Examination

The visual examination of the teeth was carried out under standardized illumination of the treatment unit (25,000 lx) using a dental mirror and air syringe.

The surfaces were assessed by modifying the classification according to Schaefer et al. [46]. Furthermore, beyond this, for comparison to the other diagnostic methods, sound teeth without visually detectable caries lesions were given the numerical value (0), surfaces with caries enamel lesions were scored using the numerical value (1), and surfaces already exhibiting caries dentinal lesions with numerical value (2).

#### 2.4.2. Bitewing Radiography

For digital bitewing radiography with a sensor (XIOSPLUS-Sensor 31.3 × 44.5 × 6.7 mm; Dentsply Sirona, Bensheim, Germany), a commercial intraoral X-ray unit (Heliodent DS, Dentsply Sirona) was used. The exposure time was 0.06 s at a cathode voltage of 60 kV and a current of 7 mA.

The caries lesions were assessed according to Marthaler, describing four different caries stages: D1 outer and D2 inner half are limited to caries enamel lesions and D3 outer and D4 inner half describing caries dentinal lesions [47]. For better comparability of the bitewing radiography data to the other diagnostic methods, the classification of Marthaler was simplified. Sound areas without radiologically detectable caries lesions were scored using a numerical value (0). Caries enamel lesions were marked with numerical values (1) and caries dentinal lesions with numerical value (2). While the greatest caries progression per tooth was assessed for the evaluation of the occlusal lesions, both the mesio-proximal and disto-proximal surfaces could be examined for proximal caries lesions. The reason for assessing the occlusal lesion according to the greatest caries progression per tooth and not per surface is the two-dimensionality of the bitewing radiography.

#### 2.4.3. Diagnocam

In addition to the visual and radiological examinations, caries diagnostics were carried out using Diagnocam (version 2.4.1.6107, KaVo). This device uses near-infrared light transillumination and operates at a wavelength of 780 nm [13]. Similar to other diagnostic methods, sound surfaces without caries lesions visible in the Diagnocam were given a numerical value (0), caries enamel lesions were marked with the numerical value (1), and surfaces showing dentinal caries with the numerical value (2).

#### 2.4.4. Trios 4

The Trios 4 (version 20.1.4, 3Shape) uses fluorescence technology with blue-violet light of a wavelength of 415 nm to detect fluorescence changes between sound and demineralized tooth surfaces [32]. In this study, occlusal and proximal caries lesions were examined. However, the manufacturer only recommends occlusal caries diagnostics.

In contrast to the Planmeca Emerald S, caries diagnostics can be performed with the Trios 4 using the same scan tip by performing a scan of the surface in the caries mode with the effect of an “overlay scan”. 

To ensure a reproducible and standardized distance of the scan head to the teeth of one centimeter, an individual placeholder was fabricated using non-reflective silicone (Plurasil Putty, Pluradent AG & Co KG, Offenbach, Germany).

According to the software, initial caries lesions are highlighted in yellow in the caries mode, while moderate to extensive lesions are displayed in red. 

For comparison of the color scale to the other diagnostic methods, sound surfaces without any color were given a numerical value (0), yellow areas that showed caries enamel lesions were scored with the numerical value (1), and red areas that showed dentinal caries were scored with the numerical value (2). 

#### 2.4.5. iTero Element 5D

In addition to the Trios 4, teeth were also assessed using the iTero Element 5D (version 2.6.3.356, Align Technology). This intraoral scanner is based on near-infrared transillumination (NIRI) technology. According to the manufacturer’s information, enamel that is illuminated with light in the near-infrared range (wavelength 850 nm) appears darkly translucent, whereas dentin or caries lesions appear less translucent and brighter [48]. Similar to Trios 4, the same scanning tip for capturing 3D images and caries diagnostics is provided.

Align Technology provides a classification into lesion grades (0) to (3) for proximal caries lesions: sound tissue (0), enamel lesions (1), lesions up to the enamel–dentin interface (2), and dentin lesions that may already appear cavitated (3). For better comparability of data to the other diagnostic methods, in this study, a distinction was made between sound surfaces (0), enamel lesions (1), and dentin lesions (2). Although the manufacturer only recommended proximal caries diagnosis with the iTero Element 5D, occlusal and proximal caries lesions were examined in this study.

#### 2.4.6. Planmeca Emerald S

In contrast to the Trios 4 and iTero Element 5D, the Planmeca Emerald S (version 6.0.1.812, Planmeca) requires a separate scan tip for caries diagnostics, or a “cariosity tip”, provided by the manufacturer for occlusal and proximal caries diagnostics. Planmeca Emerald S uses near-infrared technology, also found in the Diagnocam and the iTero Element 5D. In contrast to these, Planmeca utilizes a shorter wavelength of 727 nm. The manufacturer does not classify the lesion grades for either proximal or occlusal lesions. Therefore, the lesion was scored as follows: absence of a translucent spot (0), caries lesions limited to the enamel (1), and lesions in the dentin (2).

### 2.5. Reference Dataset

After completion of the six diagnostic methods, the reference datasets were generated by an independent institute (Werth Messtechnik) using micro-computed tomography (µ-CT, TomoScope XS-FOV, Werth Messtechnik). The exposure time was 250 ms with 1200 exposures at 360° at a voltage of 130 kV and a power of 100 W. An average resolution of 2950 × 2230 pixels was selected, and the models were scanned in a standardized manner with 2950 slices from the root tip to the occlusal surface. µ-CT provided a structural resolution of 50 µm voxel size and captured a measurement volume of 90 mm height/diameter and a diameter/height of 120 mm.

Raw data were edited to the area of interest using the corresponding software Win-Werth 3D-Grafik (Werth Messtechnik) and imported into the 3D imaging software Image J (v1.53e, Wayne Rasband, National Institute of Health, Bethesda, MD, USA). First, the quantitative measurement of the caries depth was carried out according to the caries extension (CE) index described by Kühnisch et al. [49]. Subsequently, the numerical values were classified into the semi-quantitative classification of Marthaler [47], which was already used for the “bitewing radiography” diagnostic method. Finally, for comparison with the six investigated diagnostic methods, sound areas without detectable caries lesions on µ-CT were scored with the numerical value (0), D1 and D2 lesions were summarized as enamel lesions (1), and areas with D3 and D4 lesions were scored as dentinal lesions (2). 

### 2.6. Statistical Analysis

IBM SPSS Statistics (version 27, IBM, Armonk, NY, USA) was used for the statistical analysis.

Given that this was the design of a pilot study in the preparation of a clinical trial, no sample size calculation was possible in advance. However, a post hoc precision analysis for each caries diagnostic method was performed according to the following formula of Schwarze [50]:

$$precision\;\left[\varepsilon \right]=\sqrt{\frac{z^{2}*P*Q}{n}}$$

*z* = 1.96 by alpha error of 5%;

*P* = measured sensitivity or specificity;

*Q* = 1-sensitivity or 1-specificity;

*n* = total number of sound areas or total number of areas with caries lesions.

The results are presented in the Appendix A (Table A1).

#### 2.6.1. Reliability

For each caries diagnostic method, agreement with the reference method µ-CT was calculated using Cohen’s kappa (ĸ). [40] The values of sound (0), enamel lesion (1), and dentinal lesion (2) were assumed to be nominally scaled categorical variables.

#### 2.6.2. Sensitivity and Specificity

To analyze the diagnostic quality of each method, the sensitivity, specificity, and area under curve (AUC) values were determined using receiver operating characteristic (ROC) curve analysis. Therefore, two thresholds were investigated:

- Threshold I: sound (0) versus overall caries (pooled data of enamel (1) and dentin caries (2));
- Threshold II: pooled data of sound (0) and enamel caries (1) versus dentin caries (2).

The higher the AUC values, the better the diagnostic quality [51].

#### 2.6.3. Logistic Regression

To investigate the deviation of each diagnostic method from the reference method µ-CT, logistic regression was performed. Therefore, the GENLIN-MIXED procedure was applied, and models were calculated with robust standard errors. To calculate the deviations of each diagnostic method in comparison to the reference method µ-CT, 0 (no deviation between reference and diagnostic method) and 1 (deviation) were defined as dependent variables.

## 3. Results

### 3.1. Reliability

For occlusal caries diagnostics in permanent teeth, the best agreement of all investigated diagnostic methods with the reference µ-CT was achieved using the Planmeca Emerald S, which displayed considerable agreement (ĸ = 0.700). Moderate agreement was demonstrated for visual examination (ĸ = 0.512), Diagnocam (ĸ = 0.500), and Trios 4 (ĸ = 0.431). Only sufficient agreement could be shown for iTero Element 5D (ĸ = 0.366) and bitewing radiography (ĸ = 0.297).

However, for proximal surfaces in permanent teeth, the gold standard bitewing radiography still exhibited the highest agreement with the reference µ-CT (ĸ = 0.643), followed by the Planmeca Emerald S (ĸ = 0.451) with moderate agreement. The iTero Element 5D (ĸ = 0.379), the Diagnocam (ĸ = 0.318), and visual examination (ĸ = 0.252) showed sufficient agreement. The Trios 4 achieved the lowest agreement (ĸ = 0.179).

In contrast to the findings for permanent teeth, visual examination presented a near-perfect agreement with the reference µ-CT (ĸ = 0.927) for the diagnosis of occlusal caries in primary teeth. Moderate agreement was demonstrated for the Trios 4 (ĸ = 0.579), bitewing radiography (ĸ = 0.471), and Diagnocam (ĸ = 0.469). The Planmeca Emerald S (ĸ = 0.402) and iTero Element 5D (ĸ = 0.324) showed sufficient agreement. 

**Figure 3:**
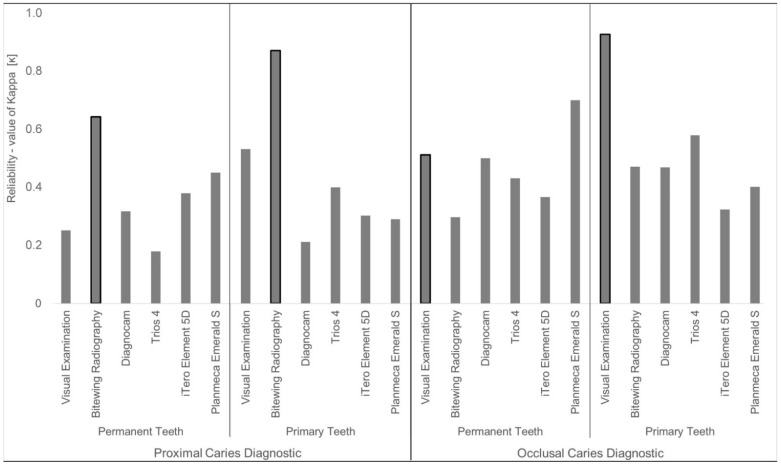
Bar graph of the reliability of the different diagnostic methods for permanent and primary teeth of proximal caries lesions and occlusal caries lesions; the respective gold standard methods are highlithed with framing of bars in bold typography.

Concerning the caries diagnostics of proximal surfaces in primary teeth, bitewing radiography showed almost perfect agreement (ĸ = 0.871), and thus the best agreement with the reference µ-CT compared to all other diagnostic methods. A moderate agreement (ĸ = 0.532) was demonstrated for visual examination, and all three Trios 4 (ĸ = 0.400), iTero Element 5D (ĸ = 0.302, Planmeca Emerald S (ĸ = 0.290), and the Diagnocam (ĸ = 0.212) achieved sufficient agreement. The reliability with values of kappa (ĸ) are displayed in Figure 3.

### 3.2. Sensitivity and Specificity

For threshold I (sound (0)) versus overall caries (pooled data of enamel (1) and dentin caries (2)), the sensitivity and specificity for the occlusal caries diagnosis in permanent teeth showed comparable results for iTero Element 5D and Trios 4, whereby Trios 4 demonstrated slightly better sensitivity and specificity values. Both intraoral scanners revealed a higher percentage of false positive than false negative diagnoses. Planmeca Emerald S exhibited the highest sensitivity, the second-highest specificity, and the highest AUC. Moreover, in terms of false negative diagnoses, Planmeca Emerald S was superior to all other diagnostic methods investigated. High sensitivity values were also found for Diagnocam, and the highest specificity was observed for visual examination.

For proximal caries diagnostics of permanent teeth, Trios 4 and iTero Element 5D showed the same sensitivity, whereby the specificity of iTero Element 5D was higher. With regard to the AUC, the Planmeca Emerald S was superior to all other diagnostic methods. The highest specificity values were found for bitewing radiography, whereas the lowest values for sensitivity were observed for visual examination. Diagnocam could demonstrate the highest sensitivity values.

**Figure 4:**
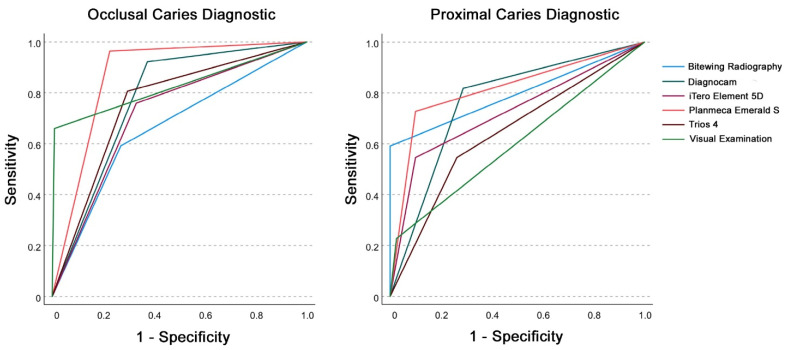
Receiver operating characteristic (ROC) curve analysis for occlusal (**left**) and proximal (**right**) caries diagnostics for the investigated diagnostic methods in permanent teeth with threshold I (sound (0)) versus overall caries (pooled data of enamel (1) and dentin caries (2)).

AUC values for IOS caries diagnostic methods in permanent teeth ranged between 0.7 and >0.8 and were classified as acceptable to excellent according to Hosmer et al. [51], except for Trios 4 in proximal lesions (Table 1 and Figure 4).

As in the case of reliability, the diagnostic methods also differed in the analysis of sensitivity and specificity between the permanent and primary teeth. For occlusal caries diagnostics in primary teeth, Trios 4 presented a high sensitivity comparable to visual examination and a low number of false positive and false negative diagnoses compared to other diagnostic methods. Planmeca Emerald S and iTero Element 5D showed the same results for specificity and the same percentage of false positive diagnoses, whereby Planmeca Emerald revealed better results in terms of sensitivity and the number of false negative diagnoses. Bitewing radiography showed the highest sensitivity, followed by visual examination showing slightly lower sensitivity.

For the detection of proximal caries lesions in primary teeth, Trios 4 showed comparable specificity with Planmeca Emerald as well as a high percentage of false positive diagnoses, which was only higher for bitewing radiography.

For iTero Element 5D, the lowest sensitivity and the highest number of false negative diagnoses for all diagnostic methods were detected on the proximal surfaces of primary teeth. Visual examination showed results similar to those of the Trios 4. Both methods are characterized by high specificity and low sensitivity. In addition, both methods had a lower percentage of false positive than false negative diagnoses.

**Figure 5:**
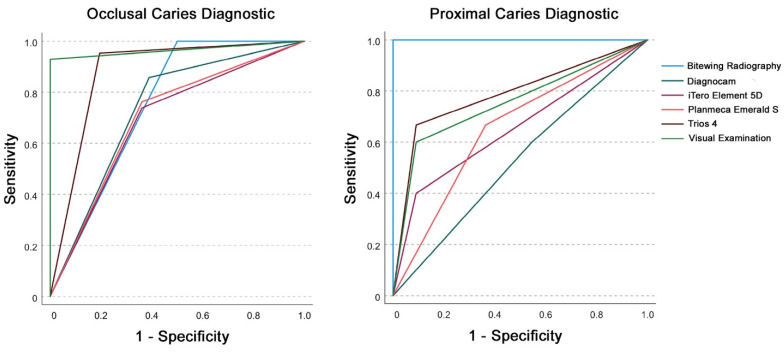
Receiver operating characteristic (ROC) curve analysis for occlusal (**left**) and proximal (**right**) caries diagnostic for the investigated diagnostic methods in primary teeth with threshold I (sound (0)) versus overall caries (pooled data of enamel (1) and dentin caries (2)).

Comparable findings could be shown in primary teeth for occlusal and proximal lesions with AUC values between 0.7 and > 0.8 (Table 1 and Figure 5).

Regarding threshold II (pooled data of sound (0) and enamel caries (1) versus dentin caries (2)), our data revealed, for occlusal caries diagnosis in permanent teeth, comparable results to threshold I, showing that Planmeca Emerald S also exhibited the highest sensitivity and highest AUC value. Furthermore, Diagnocam showed high sensitivity and AUC values. For proximal caries diagnostics of permanent teeth, bitewing radiography demonstrated superior data compared to threshold I. Planmeca Emerald S only showed a specificity of 0.6 following Diagnocam (0.7), but nevertheless both diagnostic methods have comparable AUC values of 0.7, indicating an acceptable diagnostic quality.

**Figure 6:**
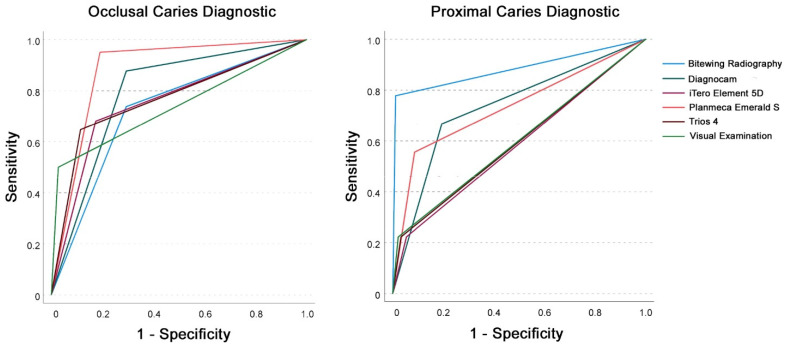
Receiver operating characteristic (ROC) curve analysis for occlusal (**left**) and proximal (**right**) caries diagnostics for the investigated diagnostic methods in permanent teeth with threshold II (sound/enamel caries versus dentin caries).

**Figure 7:**
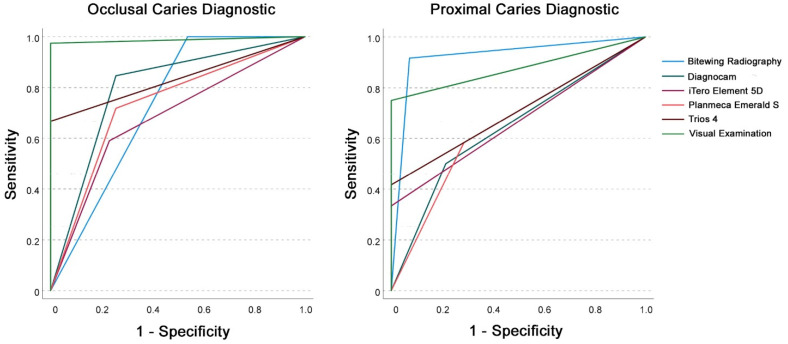
Receiver operating characteristic (ROC) curve analysis for occlusal (**left**) and proximal (**right**) caries diagnostics for the investigated diagnostic methods in primary teeth with threshold II (sound/enamel caries versus dentin caries).

In primary teeth for occlusal lesions regarding sensitivity, bitewing radiography was superior to all other diagnostic methods, with Planmeca Emerald S demonstrating the highest sensitivity of all IOS. AUC values for IOS could be demonstrated at 0.7–0.8, showing excellent diagnostic quality for Trios 4, with an AUC value of 0.8, but demonstrating visual examination to be the superior diagnostic method, with an AUC value of approximately 1.0. For the detection of proximal lesions in primary teeth, bitewing radiography showed the highest sensitivity, followed by visual examination. All other diagnostic methods, including IOS, revealed worse values regarding sensitivity (Table 2 and Figure 6 and Figure 7).

### 3.3. Logistic Regression

Data from all caries diagnostic methods were compared to the reference µ-CT. For occlusal caries diagnostics in permanent teeth, visual examination showed the lowest mean deviation on sound surfaces (1 ± 9.3%). On surfaces with caries, the Planmeca Emerald S revealed the lowest mean deviation (5 ± 21.7%). 

With regard to the diagnosis of proximal caries in permanent teeth, all diagnostic methods presented the highest mean deviation for enamel lesions, except for the Trios 4 and iTero Element 5D.

Concerning the diagnosis of occlusal caries in primary teeth, visual examination showed the lowest mean deviation (0 ± 0.0%) on sound surfaces. On surfaces with caries, bitewing radiography presented the lowest mean deviation (0 ± 0.0%) from the reference method µ-CT. 

Except for Diagnocam, all diagnostic methods for proximal lesions in primary teeth revealed the greatest mean deviation on surfaces with enamel caries. The lowest mean deviation on sound surfaces (0 ± 0.0%) and on surfaces with dentinal caries (8 ± 28.9%) was demonstrated for bitewing radiography.

The descriptive results, including correlation to the reference method µ-CT for all caries diagnostic methods in permanent and primary teeth, are presented in the Appendix A (Table A2).

**Figure 8:**
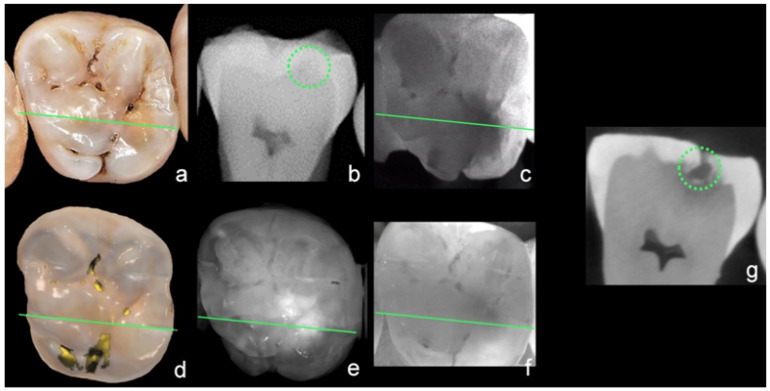
Example of all investigated caries diagnostic methods: visual examination (**a**), bitewing radiography (**b**), Diagnocam (**c**), Trios 4 (**d**), iTero Element 5D (**e**), Planmeca Emerald S (**f**), and µ-CT reference (**g**); green line shows plane of µ-CT, green circle marks caries lesion in sectional image.

Figure 8 shows an example of all caries diagnostic methods used in this study.

## 4. Discussion

In clinical practice, the detection of non-cavitated caries is challenging [13]. Furthermore, the monitoring of initial proximal lesions in the enamel is difficult only by visual examination without ionizing radiation [33]. Therefore, additional caries diagnostic tools integrated into the commercially available intraoral scanners might be a promising alternative to the currently established methods.

Current studies have demonstrated a wide variation in routine caries diagnosis [16,17]. Even when using established criteria for reporting, divergent clinical findings are reported [18]. Therefore, the training and calibration of obvious pathological findings was conducted in advance of this study. Only one examiner investigated all six diagnostic methods. Following Michou et al. [31] and Metzger et al. [33], only posterior teeth were analyzed without restoration. In addition to permanent teeth, primary molars were included in the present study as well. The caries diagnostic tools for intraoral scanners require light transmission or fluorescence technology. This is the reason that caries diagnosis in restored teeth may not be possible [52]. Thus, intraoral scanners for caries diagnostics could be a viable diagnostic alternative, especially in pediatric dentistry. Since a different hard tissue structure is described in primary and permanent teeth [53,54], we decided to investigate both dentitions in this study. To obtain basic information about the performance of caries diagnostics with intraoral scanners for occlusal and proximal caries lesions, each method was systematically analyzed separately. However, the authors are aware of the fact that, in a clinical setting, a combination of different methods are often applied and are recommended before invasive treatment of non-cavitated early caries lesions [12,20,25,26,27].

Currently, high-resolution µ-CT is an established alternative as a reference method for occlusal and proximal caries lesions instead of histology, which always requires the sectioning of teeth for investigation [37,38]. This bears the risk that the full extent of the lesion is not detected, as the teeth are not examined in total, but only in suspicious caries areas. In particular, non-cavitated lesions may be overlooked. Technically, sectioning can cause damage to the teeth [55,56], leading to the possibility of falsified results.

The potential of the Planmeca Emerald S in caries diagnostics for permanent teeth was clearly demonstrated in our study. Planmeca Emerald S demonstrated superior results for occlusal caries diagnostics in permanent dentition compared to the established gold standard visual examination. Even though the gold standard bitewing radiography exhibited the best results for proximal caries diagnostics in permanent dentition, Planmeca Emerald S showed comparable results to bitewing radiography. This aspect could be presented for both evaluated thresholds, with the fact that bitewing radiography was superior in threshold II, but nevertheless Planmeca Emerald S proved to show an acceptable AUC value, meaning it is an acceptable diagnostic tool.

However, different results were found among permanent and primary teeth. For the diagnosis of occlusal caries lesions in primary dentition, the gold standard visual examination exhibited the best results, showing the highest AUC value followed by Trios 4. Therefore, the Trios 4 might also be a viable supplementary caries diagnostic method in addition to visual examination, especially on occlusal surfaces of the primary dentition. Furthermore, the visualization of caries lesions on the screen may increase the patients’ treatment acceptance for invasive interventions, if necessary. This aspect could also be confirmed for Trios 4 in both thresholds, whereby in threshold II, Trios 4 was even considered to be superior to threshold I. Regarding the diagnosis of proximal lesions in primary teeth, bitewing radiography was still shown to be indispensable for treatment decisions and should be a permanent part of the diagnostic process, which can be underlined by both results regarding threshold I and II, showing low AUC values for all other examined caries methods.

Furthermore, the Trios 4 showed higher AUC values on occlusal surfaces compared to the proximal surfaces of the permanent dentition. This can be explained by the current scan tip, which, according to the manufacturer, only allows the detection of occlusal caries. Therefore, the results of proximal caries diagnostics must be interpreted carefully. 

In the study by Michou et al. [31], the “f4 function” on occlusal surfaces of the permanent dentition, with a sum of sensitivity and specificity of 160–184% (depending on the degree of lesion), showed the best overall performance. All the functions investigated for the Trios 3-based prototype with caries diagnosis performed well. These results are in accordance with our data for occlusal caries diagnostics using Trios 4 in permanent teeth, showing an acceptable AUC value. 

In contrast to Trios 4, Align Technology released the iTero Element 5D only for proximal surfaces and provided a classification for caries lesions. Therefore, the results of occlusal caries diagnostics must also be interpreted with care. This guideline was confirmed in our study, in which the iTero Element 5D showed better specificity values and greater reliability on proximal surfaces (SP = 90%) than occlusal surfaces (SP = 67%) in the permanent dentition. Metzger et al. [33] also investigated the caries diagnostic function on proximal surfaces of the permanent dentition in their clinical study, showing similar results for caries lesions in the enamel (SE = 51.6%, SP = 90.4%) compared with those in our study (SE = 55%, SP = 90%). While the Cohen’s kappa value was lower than in the present study, according to the classification of Landis and Koch [40], these data have to be interpreted as sufficient. Although a direct comparison of the results remains difficult, the reliability determined by Metzger et al. is similar to our findings [33].

For the Planmeca Emerald S, the highest AUC value of all examination methods could be determined on occlusal surfaces (0.87) in the permanent dentition. We hypothesize that the better results of Planmeca Emerald S compared to iTero Element 5D and Diagnocam can be explained by the different wavelengths applied. Although the three methods are all based on the same functional principle of near-infrared transillumination, the Planmeca Emerald uses the lowest wavelength (727 nm), followed by Diagnocam (780 nm) and iTero Element 5D (850 nm). In this study, the reliability is inversely related to the wavelength of near-infrared transillumination.

Overall, the results of the present study demonstrated that none of the diagnostic methods can be universally applied to occlusal and proximal surfaces in both dentitions. However, the Planmeca Emerald 5 and Trios 4 seem to be suitable tools for clinical practice. The use of the Trios 4 as a basic instrument, which only needs to be supplemented by bitewing radiographs in the case of unclear findings, would be desirable, particularly in pediatric patients. Among children and adolescents, for whom regular bitewing radiographs may be contraindicated with regard to X-ray exposure, a radiation-free intraoral scan could be an additional helpful tool for routine dental examinations. 

Concerning the limitations of this study, we have to point out that the intraoral scanners is a light optical system. Therefore, the detection of subgingival caries lesions and teeth with restorations with secondary caries is not possible. Thus, the application field is limited in these cases. Furthermore, regarding enamel caries, all the examined diagnostic methods may have an increased error rate. For clinical practice, it would be interesting to measure the required time for each caries diagnostic method. However, we decided not to investigate this aspect because results might be misleading due to the in vitro study design in a phantom head without any saliva, movement, and patient-specific influencing factors. Importantly, the results of the diagnostics of dentin caries should be interpreted with care because of the low number of lesions.

For a useful implementation of caries diagnostics with intraoral scanners in routine patient care, it would be desirable if the intraoral scanner software would automatically mark conspicuous areas in the intraoral scan with the help of artificial intelligence. This has already been described in the field of reporting radiographs [57], clinical photos [58], or NIRI images [59]. Finally, these results must be clarified by a prospective clinical trial, which is in preparation.

## 5. Conclusions

Within the limitations of this in vitro study, no universal diagnostic method could be identified in general as suitable for occlusal and proximal caries diagnostics in both dentitions.

- Planmeca Emerald S demonstrated better results for occlusal caries diagnostics in permanent dentition compared to established gold standard visual examination.
- For diagnosis of occlusal caries lesions in primary dentition, the gold standard visual examination exhibited the best results.
- Concerning proximal caries lesions, the gold standard bitewing radiography is still not substitutable, but in permanent dentition, Planmeca Emerald S showed even better results regarding AUC value than radiography.

Overall, caries diagnostics with intraoral scanners seems to be an interesting tool that should be further investigated in clinical studies.

## Figures and Tables

**Table 1:** Caries diagnostic methods regarding sensitivity, specificity, and area under curve (AUC) values with receiver operating characteristic (ROC) curve analysis for threshold I (sound (0)) versus overall caries (pooled data of enamel (1) and dentin caries (2)).

		Permanent Teeth			Primary Teeth		
Region of Interest	Diagnostic Methods	Sensitivity	Specificicy	AUC	Sensitivity	Specificicy	AUC
Occlusal Surfaces	Visual Examination	0.660	0.991	0.825	0.929	1.000	0.964
	Bitewing Radiography	0.592	0.730	0.661	1.000	0.500	0.750
	Diagnocam	0.921	0.626	0.774	0.857	0.611	0.734
	Trios 4	0.806	0.704	0.755	0.952	0.806	0.879
	iTero Element 5D	0.759	0.670	0.714	0.738	0.639	0.688
	Planmeca Emerald S	0.963	0.774	0.869	0.762	0.639	0.700
Proximal Surfaces	Visual Examination	0.227	0.750	0.601	0.600	0.909	0.755
	Bitewing Radiography	0.591	1.000	0.795	1.000	1.000	1.000
	Diagnocam	0.818	0.712	0.765	0.600	0.455	0.527
	Trios 4	0.545	0.738	0.641	0.667	0.909	0.788
	iTero Element 5D	0.545	0.900	0.723	0.400	0.909	0.655
	Planmeca Emerald S	0.727	0.900	0.814	0.667	0.636	0.652

**Table 2:** Caries diagnostic methods regarding sensitivity, specificity, and area under curve (AUC) values with receiver operating characteristic (ROC) curve analysis for threshold II (sound/enamel caries versus dentin caries).

		Permanent Teeth			Primary Teeth		
Region of Interest	Diagnostic Methods	Sensitivity	Specificicy	AUC	Sensitivity	Specificicy	AUC
Occlusal Surfaces	Visual Examination	0.500	0.973	0.736	0.974	1.000	0.987
	Bitewing Radiography	0.738	0.707	0.722	1.000	0.462	0.731
	Diagnocam	0.877	0.707	0.792	0.846	0.744	0.795
	Trios 4	0.648	0.886	0.767	0.667	1.000	0.833
	iTero Element 5D	0.680	0.826	0.753	0.590	0.769	0.679
	Planmeca Emerald S	0.951	0.810	0.880	0.718	0.744	0.731
Proximal Surfaces	Visual Examination	0.222	0.978	0.600	0.750	1.000	0.875
	Bitewing Radiography	0.778	0.989	0.884	0.917	0.929	0.923
	Diagnocam	0.667	0.806	0.737	0.500	0.786	0.643
	Trios 4	0.222	0.968	0.595	0.417	1.000	0.708
	iTero Element 5D	0.222	0.946	0.584	0.333	1.000	0.667
	Planmeca Emerald S	0.556	0.914	0.735	0.583	0.714	0.649

## Data Availability

The datasets of this article are available from the corresponding author on a reasonable request.

## Appendix A

**Table A1:** Precision of sensitivity and specificity in permanent and primary teeth for the different diagnostic methods (respective gold standard diagnostic method is highlighted in bold typography).

	Region of Interest	Diagnostic Methods	Precision (ɛ)	
			For Sensitivity (%)	For Specificity (%)
Permanent Teeth	Occlusal Surfaces	Visual Examination	±7	±2
		Bitewing Radiography	±7	±8
		Diagnocam	±4	±9
		Trios 4	±6	±8
		iTero Element 5D	±6	±9
		Planmeca Emerald S	±3	±8
	Proximal Surfaces	Visual Examination	±18	±3
		Bitewing Radiography	±21	±0
		Diagnocam	±16	±10
		Trios 4	±21	±10
		iTero Element 5D	±21	±7
		Planmeca Emerald S	±19	±7
Primary Teeth	Occlusal Surfaces	Visual Examination	±8	±0
		Bitewing Radiography	±0	±16
		Diagnocam	±10	±16
		Trios 4	±7	±13
		iTero Element 5D	±13	±16
		Planmeca Emerald S	±13	±16
	Proximal Surfaces	Visual Examination	±25	±17
		Bitewing Radiography	±0	±0
		Diagnocam	±25	±29
		Trios 4	±24	±17
		iTero Element 5D	±25	±17
		Planmeca Emerald S	±24	±28

**Table A2:** Deviations (%) of all methods from the reference method µ-CT on occlusal and proximal surfaces of permanent and primary teeth for the different diagnostic methods.

				Permanent Teeth			Primary Teeth		
Region of Interst	Diagnostic Methods	µ-CT Groups		Mean (%)	SD (%)	N	Mean (%)	SD (%)	N
Occlusal Surfaces	Visual Examination	Sound	(0)	1	9.3	115	0	0	36
		Enamel lesion	(1)	51	50.4	69	67	57.7	3
		Dentinal lesion	(2)	50	50.2	122	3	16.0	39
	Bitewing Radiography	Sound	(0)	27	44.6	115	50	50.7	36
		Enamel lesion	(1)	100	0.0	69	100	0	3
		Dentinal lesion	(2)	26	44.2	122	0	0	39
	Diagnocam	Sound	(0)	37	48.6	115	39	49.4	36
		Enamel lesion	(1)	58	49.7	69	100	0	3
		Dentinal lesion	(2)	12	33.0	122	15	36.6	39
	Trios 4	Sound	(0)	30	45.8	115	19	40.1	36
		Enamel lesion	(1)	55	50.1	69	33	57.7	3
		Dentinal lesion	(2)	35	48.0	122	33	47.8	39
	iTero Element 5D	Sound	(0)	33	47.2	115	36	48.7	36
		Enamel lesion	(1)	71	45.7	69	67	57.7	3
		Dentinal lesion	(2)	32	46.8	122	41	49.8	39
	Planmeca Emerald S	Sound	(0)	23	42.0	115	36	48.7	36
		Enamel lesion	(1)	39	49.2	69	67	57.7	3
		Dentinal lesion	(2)	5	21.7	122	28	45.6	39
Proximal Surfaces	Visual Examination	Sound	(0)	3	15.7	80	9	30.2	11
		Enamel lesion	(1)	85	37.6	13	100	0	3
		Dentinal lesion	(2)	78	44.1	9	25	45.2	12
	Bitewing Radiography	Sound	(0)	0	0	80	0	0	11
		Enamel lesion	(1)	69	48.0	13	33	57.7	3
		Dentinal lesion	(2)	22	44.1	9	8	28.9	12
	Diagnocam	Sound	(0)	29	45.5	80	55	52.2	11
		Enamel lesion	(1)	62	50.6	13	33	57.7	3
		Dentinal lesion	(2)	33	50.0	9	50	52.2	12
	Trios 4	Sound	(0)	26	44.3	80	9	30.2	11
		Enamel lesion	(1)	62	50.6	13	67	57.7	3
		Dentinal lesion	(2)	78	44.1	9	58	51.5	12
	iTero Element 5D	Sound	(0)	10	30.2	80	9	30.2	11
		Enamel lesion	(1)	54	51.9	13	67	57.7	3
		Dentinal lesion	(2)	78	44.1	9	67	49.2	12
	Planmeca Emerald S	Sound	(0)	10	30.2	80	36	50.5	11
		Enamel lesion	(1)	69	48.0	13	67	57.7	3
		Dentinal lesion	(2)	44	52.7	9	42	51.5	12

## References

[1] Schlenz M.A., Schlenz M.B., Wöstmann B., Jungert A., Ganss C. (2022). Intraoral scanner-based monitoring of tooth wear in young adults: 12-month results. Clin. Oral Investig..

[2] Mehl A., Bosch G., Fischer C., Ender A. (2017). In vivo tooth-color measurement with a new 3D intraoral scanning system in comparison to conventional digital and visual color determination methods. Int. J. Comput. Dent..

[3] Daher R., Ardu S., Vjero O., Krejci I. (2018). 3D Digital Smile Design with a Mobile Phone and Intraoral Optical Scanner. Compend. Contin. Educ. Dent..

[4] Yun D., Choi D.S., Jang I., Cha B.K. (2018). Clinical application of an intraoral scanner for serial evaluation of orthodontic tooth movement: A preliminary study. Korean J. Orthod..

[5] Suese K. (2020). Progress in digital dentistry: The practical use of intraoral scanners. Dent. Mater. J..

[6] Disease G.B.D., Injury I., Prevalence C. (2018). Global, regional, and national incidence, prevalence, and years lived with disability for 354 diseases and injuries for 195 countries and territories, 1990–2017: A systematic analysis for the Global Burden of Disease Study 2017. Lancet.

[7] WHO Oral Health. https://www.who.int/news-room/fact-sheets/detail/oral-health.

[8] Pitts N. (2009). Detection, Assessment, Diagnosis and Monitoring of Caries.

[9] Kühnisch J., Ekstrand K.R., Pretty I., Twetman S., van Loveren C., Gizani S., Spyridonos Loizidou M. (2016). Best clinical practice guidance for management of early caries lesions in children and young adults: An EAPD policy document. Eur. Arch. Paediatr. Dent..

[10] Schwendicke F., Splieth C., Breschi L., Banerjee A., Fontana M., Paris S., Burrow M.F., Crombie F., Page L.F., Gaton-Hernandez P. (2019). When to intervene in the caries process?. An expert Delphi consensus statement. Clin. Oral Investig..

[11] Borzabadi-Farahani A., Eslamipour F., Asgari I. (2011). Association between orthodontic treatment need and caries experience. Acta Odontol. Scand..

[12] Souza J.F., Boldieri T., Diniz M.B., Rodrigues J.A., Lussi A., Cordeiro R.C. (2013). Traditional and novel methods for occlusal caries detection: Performance on primary teeth. Lasers Med. Sci..

[13] Abogazalah N., Ando M. (2017). Alternative methods to visual and radiographic examinations for approximal caries detection. J. Oral Sci..

[14] Bader J.D., Shugars D.A., Bonito A.J. (2002). A systematic review of the performance of methods for identifying caries lesions. J. Public Health Dent..

[15] Pitts N.B., Stamm J.W. (2004). International Consensus Workshop on Caries Clinical Trials (ICW-CCT)—Final consensus statements: Agreeing where the evidence leads. J. Dent. Res..

[16] Neuhaus K.W., Longbottom C., Ellwood R., Lussi A. (2009). Novel lesion detection aids. Monogr. Oral Sci..

[17] Novaes T.F., Matos R., Braga M.M., Imparato J.C., Raggio D.P., Mendes F.M. (2009). Performance of a pen-type laser fluorescence device and conventional methods in detecting approximal caries lesions in primary teeth—In vivo study. Caries Res..

[18] Braga M.M., Morais C.C., Nakama R.C., Leamari V.M., Siqueira W.L., Mendes F.M. (2009). In vitro performance of methods of approximal caries detection in primary molars. Oral Surg. Oral Med. Oral Pathol. Oral Radiol. Endod..

[19] Shi X.Q., Li G. (2009). Detection accuracy of approximal caries by black-and-white and color-coded digital radiographs. Oral Surg. Oral Med. Oral Pathol. Oral Radiol. Endod..

[20] Huth K.C., Lussi A., Gygax M., Thum M., Crispin A., Paschos E., Hickel R., Neuhaus K.W. (2010). In vivo performance of a laser fluorescence device for the approximal detection of caries in permanent molars. J. Dent..

[21] Schwendicke F., Tzschoppe M., Paris S. (2015). Radiographic caries detection: A systematic review and meta-analysis. J. Dent..

[22] Fried D., Featherstone J.D., Darling C.L., Jones R.S., Ngaotheppitak P., Buhler C.M. (2005). Early caries imaging and monitoring with near-infrared light. Dent. Clin. N. Am..

[23] Kühnisch J., Sochtig F., Pitchika V., Laubender R., Neuhaus K.W., Lussi A., Hickel R. (2016). In vivo validation of near-infrared light transillumination for interproximal dentin caries detection. Clin. Oral Investig..

[24] Alamoudi N.M., Khan J.A., El-Ashiry E.A., Felemban O.M., Bagher S.M., Al-Tuwirqi A.A. (2019). Accuracy of the DIAGNOcam and bitewing radiographs in the diagnosis of cavitated proximal caries lesions in primary molars. Niger. J. Clin. Pract..

[25] Sochtig F., Hickel R., Kühnisch J. (2014). Caries detection and diagnostic with near-infrared light transillumination: Clinical experiences. Quintessence Int..

[26] Gomez J. (2015). Detection and diagnosis of the early caries lesion. BMC Oral Health.

[27] Mileman P.A., van der Weele L.T. (1990). Accuracy in radiographic diagnosis: Dutch practitioners and dental caries. J. Dent..

[28] De Araujo F.B., Rosito D.B., Toigo E., dos Santos C.K. (1992). Diagnosis of approximal caries: Radiographic versus clinical examination using tooth separation. Am. J. Dent..

[29] Janjic Rankovic M., Kapor S., Khazaei Y., Crispin A., Schuler I., Krause F., Ekstrand K., Michou S., Eggmann F., Lussi A. (2021). Systematic review and meta-analysis of diagnostic studies of proximal surface caries. Clin. Oral Investig..

[30] Jablonski-Momeni A., Moos J., Sakhaei Manesh V., Stoll R. (2018). Diagnostic Accuracy of a Bioluminescence System for the Assessment of Caries Activity on Occlusal Surfaces. Caries Res..

[31] Michou S., Benetti A.R., Vannahme C., Hermannsson P.G., Bakhshandeh A., Ekstrand K.R. (2020). Development of a Fluorescence-Based Caries Scoring System for an Intraoral Scanner: An In Vitro Study. Caries Res..

[32] Michou S., Lambach M.S., Ntovas P., Benetti A.R., Bakhshandeh A., Rahiotis C., Ekstrand K.R., Vannahme C. (2021). Automated caries detection in vivo using a 3D intraoral scanner. Sci. Rep..

[33] Metzger Z., Colson D.G., Bown P., Weihard T., Baresel I., Nolting T. (2022). Reflected near-infrared light versus bite-wing radiography for the detection of proximal caries: A multicenter prospective clinical study conducted in private practices. J. Dent..

[34] Litzenburger F., Heck K., Kaisarly D., Kunzelmann K.H. (2022). Diagnostic validity of early proximal caries detection using near-infrared imaging technology on 3D range data of posterior teeth. Clin. Oral Investig..

[35] Lederer A., Kunzelmann K.H., Heck K., Hickel R., Litzenburger F. (2019). In-vitro validation of near-infrared reflection for proximal caries detection. Eur. J. Oral Sci..

[36] Litzenburger F., Lederer A., Kollmuss M., Hickel R., Kunzelmann K.H., Heck K. (2020). Near-infrared transillumination with high dynamic range imaging for occlusal caries detection in vitro. Lasers Med. Sci..

[37] Mitropoulos P., Rahiotis C., Stamatakis H., Kakaboura A. (2010). Diagnostic performance of the visual caries classification system ICDAS II versus radiography and micro-computed tomography for proximal caries detection: An in vitro study. J. Dent..

[38] Soviero V.M., Leal S.C., Silva R.C., Azevedo R.B. (2012). Validity of MicroCT for in vitro detection of proximal caries lesions in primary molars. J. Dent..

[39] Pieper K., Blumenstein A. (1993). Die Zahnmedizinische Untersuchung im Rahmen der Gruppenprophylaxe: Ein Leitfaden für die EDV-Gestützte Erfolgskontrolle.

[40] Landis J.R., Koch G.G. (1977). The measurement of observer agreement for categorical data. Biometrics.

[41] Müller P., Ender A., Joda T., Katsoulis J. (2016). Impact of digital intraoral scan strategies on the impression accuracy using the trios pod scanner. Quintessence Int..

[42] (2003). Dental Materials—Testing of Adhesion to Tooth Structure.

[43] Arakida T., Kanazawa M., Iwaki M., Suzuki T., Minakuchi S. (2018). Evaluating the influence of ambient light on scanning trueness, precision, and time of intra oral scanner. J. Prosthodont. Res..

[44] Wesemann C., Kienbaum H., Thun M., Spies B.C., Beuer F., Bumann A. (2021). Does ambient light affect the accuracy and scanning time of intraoral scans?. J. Prosthet. Dent..

[45] Schlenz M.A., Vogler J.A.H., Schmidt A., Rehmann P., Wöstmann B. (2020). Chairside measurement of the marginal and internal fit of crowns: A new intraoral scan-based approach. Clin. Oral Investig..

[46] Schaefer G., Pitchika V., Litzenburger F., Hickel R., Kühnisch J. (2018). Evaluation of occlusal caries detection and assessment by visual inspection, digital bitewing radiography and near-infrared light transillumination. Clin. Oral Investig..

[47] Marthaler T.M. (1966). A standardized system of recording dental conditions. Helv. Odontol. Acta.

[48] Align Technology B.V. iTero Element 5D—Benutzerhandbuch. https://storagy-itero-production-eu.s3.amazonaws.com/download/de-de/iTero-Element-5D-User-Manual.pdf.

[49] Kühnisch J., Bucher K., Henschel V., Albrecht A., Garcia-Godoy F., Mansmann U., Hickel R., Heinrich-Weltzien R. (2011). Diagnostic performance of the universal visual scoring system (UniViSS) on occlusal surfaces. Clin. Oral Investig..

[50] Schwarze J. (1997). Wahrscheinlichkeitsrechnung und Induktive Statistik.

[51] Hosmer D.W., Lemeshow S. (2000). Applied Logistic Regression.

[52] Abdelaziz M., Krejci I. (2015). DIAGNOcam—A Near Infrared Digital Imaging Transillumination (NIDIT) technology. Int. J. Esthet. Dent..

[53] Mortimer K.V. (1970). The relationship of deciduous enamel structure to dental disease. Caries Res..

[54] Uhlmann U. (2019). Kinderzahnheilkunde—Grundlagen für die Tägliche Praxis.

[55] Boca C., Truyen B., Henin L., Schulte A.G., Stachniss V., De Clerck N., Cornelis J., Bottenberg P. (2017). Comparison of micro-CT imaging and histology for approximal caries detection. Sci. Rep..

[56] Özkan G., Kanli A., Baseren N.M., Arslan U., Tatar I. (2015). Validation of micro-computed tomography for occlusal caries detection: An in vitro study. Braz. Oral Res..

[57] Khanagar S.B., Al-Ehaideb A., Maganur P.C., Vishwanathaiah S., Patil S., Baeshen H.A., Sarode S.C., Bhandi S. (2021). Developments, application, and performance of artificial intelligence in dentistry—A systematic review. J. Dent. Sci..

[58] Kühnisch J., Meyer O., Hesenius M., Hickel R., Gruhn V. (2022). Caries Detection on Intraoral Images Using Artificial Intelligence. J. Dent. Res..

[59] Casalegno F., Newton T., Daher R., Abdelaziz M., Lodi-Rizzini A., Schurmann F., Krejci I., Markram H. (2019). Caries Detection with Near-Infrared Transillumination Using Deep Learning. J. Dent. Res..

